# Moderately decreased maternal dietary energy intake during pregnancy reduces fetal skeletal muscle mitochondrial biogenesis in the pigs

**DOI:** 10.1186/s12263-016-0535-1

**Published:** 2016-06-23

**Authors:** Tiande Zou, Bing Yu, Jie Yu, Xiangbing Mao, Ping Zheng, Jun He, Zhiqing Huang, Yue Liu, Daiwen Chen

**Affiliations:** Key Laboratory for Animal Disease-Resistant Nutrition of the Ministry of Education of China, Animal Nutrition Institute, Sichuan Agricultural University, Cheng du, China

**Keywords:** Maternal energy intake, Skeletal muscle, Mitochondrial biogenesis, Oxidative stress, Fetal pig

## Abstract

**Background:**

Mitochondria are of major importance in oocyte and early embryo, playing a key role in maintaining energy homeostasis. Epidemiological findings indicate that maternal undernutrition-induced mitochondrial dysfunction during pregnancy is associated with the development of metabolic disorders in offspring. Here, we investigated the effects of moderately decreased maternal energy intake during pregnancy on skeletal muscle mitochondrial biogenesis in fetal offspring with pig as a model.

**Methods:**

Pregnant Meishan sows were allocated to a standard-energy (SE) intake group as recommended by the National Research Council (NRC; 2012) and a low-energy (LE) intake group. Fetal umbilical vein serum and longissimus muscle samples were collected for further analysis on day 90 of pregnancy.

**Results:**

Sow and fetal weights and the concentrations of serum growth hormone (GH) and glucose were reduced in LE group. Maternal LE diet decreased the messenger RNA (mRNA) expression of genes involved in mitochondrial biogenesis and function such as peroxisome proliferator-activated receptor gamma coactivator 1α (PPARGC1A), nuclear respiratory factor 1 (NRF1), mitochondrial transcription factor A (TFAM), β subunit of mitochondrial H^+^-ATP synthase (ATB5B), sirtuin 1 (Sirt1), and citrate synthase (CS). The protein expression of PPARGC1A and Sirt1, intracellular NAD^+^-to-NADH ratio, and CS activity was reduced in LE group, and accordingly, mitochondrial DNA (mtDNA) content was decreased. Moreover, copper/zinc superoxide dismutase (CuZn-SOD) expression at both mRNA and protein levels and SOD and catalase (CAT) activities were reduced in LE group as well.

**Conclusions:**

The observed decrease in muscle mitochondrial biogenesis and antioxidant defense capacity suggests that moderately decreased maternal energy intake during pregnancy impairs mitochondrial function in fetal pigs.

## Background

Obesity, characterized by an imbalance between energy intake and expenditure, is increasing at an alarming rate in most parts of the world. In particular, human epidemiological and animal experimental studies have demonstrated that hormonal, metabolic, and nutritional disturbances at crucial prenatal time periods may also determine the predisposition to deleterious health outcomes in adult life, including obesity and associated metabolic disorders [3, 12, 20]. Thus, maternal undernutrition during pregnancy has been reported to have persistent effects on offspring metabolic energy regulatory systems [42, 45], although the underlying mechanisms of this process are still unclear.

Mitochondria are of major importance in oocyte and early embryo, playing a key role in maintaining energy homeostasis [26, 40]. Deregulation of energy homeostasis is a common underlying characteristic of metabolic syndrome, and many studies have demonstrated that prenatal maternal undernutrition programs offspring mitochondrial function in various tissues of different animal models, such as the pancreatic islet [41] and kidney [9] of rat and the skeletal muscle of sheep [17]. The skeletal muscle composes about 40–50 % of body mass and is the major site of glucose and fatty acid utilization, playing an important role in preventing obesity and type 2 diabetes (T2D) [36, 47]. There is growing evidence that increased mitochondrial function may be essential for skeletal myogenesis [33]. Moreover, mitochondrial dysfunction in the skeletal muscle is been implicated in the development of T2D [2, 19]. Studies in mice have shown that maternal exposure to a low-protein diet during pregnancy and lactation decreases mitochondrial DNA content and citrate synthase activity and leads to mitochondrial gene expression changes in the offspring liver and skeletal muscle [18, 27]. However, currently, to our knowledge, no studies have addressed the effects of maternal low-energy (LE) diets on fetal offspring skeletal muscle mitochondrial biogenesis and function.

Compared with sheep and rodents, pigs share a number of anatomical and physiological similarities with humans making them an excellent experimental model for metabolic studies [31, 39]. However, there is no data in the pig skeletal muscle on how maternal LE intake during pregnancy may affect mitochondrial number and function. In addition, prenatal skeletal muscle development is particularly susceptible to environmental factors in mammalians [8]. Thus, understanding the effect of maternal LE diet on mitochondrial biogenesis and function in the skeletal muscle of the pig fetuses may provide important insights into the fetal origins of the metabolic syndrome. In the present study, our objective was to investigate the effects of moderately decreased maternal dietary energy intake during pregnancy on skeletal muscle mitochondrial biogenesis in pig fetuses on day 90 of pregnancy.

## Methods

### Animals and diets

**Fig. 1:**
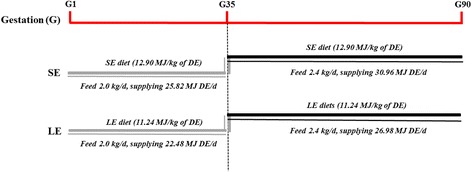
The experimental design and the timing of the different treatments and procedures. *SE* maternal standard-energy diet, *LE* maternal low-energy diet, *DE* digestible energy

The experimental procedures were approved by the Animal Care and Use Committee of Sichuan Agricultural University. Eight primiparous, purebred Meishan sows (initial body weight 72.8 ± 4.0 kg) were artificially inseminated three times, at the third observation of estrus, with the semen of purebred Meishan boars. The day of the last insemination constituted the first day of pregnancy. The pregnant sows were randomly assigned to one of the two experimental groups differing in daily digestible energy (DE) intake: standard-energy (SE) and low-energy (LE) groups. Based on the consideration that Meishan pigs, being raised with modern commercial diets composed following NRC standards, were traditionally raised on LE diets, we fed the sows in SE group with diets containing 12.90 MJ of DE/kg, while those in LE group were fed diets containing 11.24 MJ of DE/kg to mimic the energy levels in traditional diets during pregnancy. During early pregnancy (days 1 to 35 of pregnancy), sows were fed with rations of 2.0 kg/day because lower feed intake level is beneficial to embryo survival during early pregnancy [16], supplying 25.80 and 22.48 MJ DE per day for SE and LE sows, respectively. Additionally, considering the rapid increasing demand of maternal weight gain and fetal growth during mid-late pregnancy (days 36 to 90 of pregnancy) [25], sows were fed on diets with the rations of 2.4 kg/day, supplying 30.96 and 26.98 MJ DE per day for SE and LE sows, respectively (Fig. 1). The isoprotein corn-wheat bran-soybean meal-based diets were used, and the LE diet was formulated to allow the sow to ingest approximately 13 % less DE than the SE group (Table 1). Although the additional dietary fiber was added to the LE diet to decrease energy concentration, crude fiber (CF) intake was only 1.52 % higher in LE sows than in SE sows. Danielsen and Vestergaard [6] reported that even excess dietary fiber intake (CF, 4.6 vs 12.7 %) of pregnant sows influenced neither fetal survival nor litter size at birth. Consequently, it is quite unlikely in this experiment that the dietary fiber supplementation affected the fetal survival rate directly. The dietary treatments were introduced from mating to day 90 of pregnancy. During pregnancy, sows were housed in individual feed stalls in a breeding facility. Sows were fed discretely twice daily (08:00 and 14:00 h) with 50 % of the daily ration each time and had free access to drinking water.

**Table 1:** Ingredients and composition of the experimental diets (as-fed basis)

Items	SE	LE
Ingredient (%)		
Maize	45.00	45.00
Soybean meal	13.60	13.60
Wheat bran	27.80	27.80
Soybean oil	4.50	–
Wheat bran fiber	2.54	5.02
Soyabean fiber	1.10	2.17
Corn fiber	0.96	1.91
Salt	0.40	0.40
L-Lys·HCl, 75 %	0.10	0.10
L-Thr	0.10	0.10
Limestone	1.23	1.23
Monocalcium phosphate	1.99	1.99
Choline chloride, 50 %	0.14	0.14
Vitamin premix^a^	0.04	0.04
Mineral premix^b^	0.50	0.50
Total	100.00	100.00
Nutritional composition		
Digestible energy, MJ/kg	12.90	11.24
Crude protein (%)	13.92	14.35
Crude fiber (%)	4.97	6.49
Lys (%)	0.69	0.69
Met + Cys (%)	0.35	0.35
Thr (%)	0.46	0.46
Ca (%)	0.96	0.96
Available P (%)	0.48	0.48
Total P (%)	0.79	0.79

^a^Provided the following per kilogram of diet: 1.2 mg retinol, 0.02 mg cholecalciferol, 44 mg α-tocopherol, 0.5 mg phylloquinone, 1 mg thiamin, 3.75 mg riboflavin, 1 mg pyridoxine, 0.015 mg cyanocobalamin, 10 mg niacin, 12 mg pantothenic acid, 1.3 mg folic acid, and 0.2 mg biotin

^b^Provided the following per kilogram of diet: 80 mg Fe (as FeSO_4·_7H_2_O), 10 mg Cu (as CuSO_4·_5H_2_O), 100 mg Zn (as ZnSO_4·_7H_2_O), 25 mg Mn (as MnSO_4·_H_2_O), 0.15 mg Se (as Na_2_SeO_3_), and 0.14 mg I (as KI)

### Sample collection

After a 12-h overnight fast, pregnant sows of both groups (SE/LE) were weighed and anesthetized with an intramuscular injection of Zoletil (Zoletil 50, Virbac; 4 mg/kg body weight) on day 90 of pregnancy (*n* = 4/4). The maternal abdomen was opened, and the reproductive tracts were removed immediately. Blood from the umbilical vein of each fetus was collected and coagulated for 30 min and then centrifuged at 3500×*g* for 10 min to separate serum which was stored at −20 °C until analysis. The body weight of each fetus was recorded. Two female fetuses and two male fetuses, with weight close to the average level, were selected from each sow for muscle sample collection. The longissimus muscles (LMs) were collected from the fetuses, snap-frozen in liquid nitrogen, and stored at −80 °C until subsequent analysis.

### Measurement of metabolites and hormone

The malondialdehyde (MDA) concentration in the skeletal muscle was measured using an assay kit (Jiancheng Institute of Bioengineering, Nanjing, Jiangsu, China). The concentrations of triglyceride and glucose in umbilical vein serum were determined using commercial kits (Jiancheng Institute of Bioengineering, Nanjing, Jiangsu, China) according to the provided instructions. Growth hormone (GH) concentration in umbilical vein serum was measured using a commercially available radioimmunoassay kits purchased from Beijing North Institute of Biotechnology (Beijing, China). Insulin was determined with porcine ELISA kit (R&D Systems, Minneapolis, MN, USA). Sensitivities of the assays were 0.02 ng/ml and 2.15 pmol/l for GH and insulin, respectively. Intra- and inter-assay coefficients of variation were 4.3 and 6.6 % for GH and 3.9 and 7.4 % for insulin, respectively.

### Measurement of enzyme activities

The activities of superoxide dismutase (SOD), glutathione peroxidase (GPx), and catalase (CAT) in the skeletal muscle were measured using assay kits (Jiancheng Institute of Bioengineering, Nanjing, Jiangsu, China). Citrate synthase (CS) activity in the skeletal muscle was measured spectrophotometrically using a commercial kit (GenMed Scientifics Inc, USA).

### NAD^+^-to-NADH ratio measurement

The NAD^+^-to-NADH ratio in the skeletal muscle was measured using a colorimetric assay kit (BioVision, Milpitas, CA, USA) according to the manufacturer’s instructions. Briefly, muscle samples were washed with cold PBS followed homogenization using NAD^+^/NADH extraction buffer. To decompose NAD^+^, 200 μl of extract was heated to 60 °C for 30 min. Under this condition, all NAD^+^ were decomposed but keeping NADH intact. Both heated and unheated extract from each sample, together with the NADH standard solutions, were transferred into 96-well plates, added with 100 μl of NAD cycling mix into each well, mixed, and incubated at room temperature for 5 min to convert NAD^+^ to NADH. Then, 10 μl of NADH developer was added into each well and incubated at room temperature for 2 h. Read the plate at OD 450 nm. The amount of NAD^+^ from each sample was calculated as total NAD (values from the unheated extracts) minus NADH (values from the heated extracts) and then divided by the protein concentration.

### Mitochondrial DNA copy number

Total DNA was extracted from LM muscle using QIAamp DNA extraction kit (QIAGEN, Valencia, CA, USA). The mitochondrial DNA (mtDNA) copy number was determined using quantitative real-time PCR as previously described with some modifications [5]. mtDNA was amplified using primers specific for the mitochondrial cytochrome b (*MT-CYB*) and normalized to genomic DNA by amplification of the *18S rRNA*. The sequence of primers was presented in Table 2.

**Table 2:** Primer sequences of the target and reference genes

Gene	Primer sequence (5′–3′)	Product size (bp)	GeneBank no.
*PPARGC1A*	F: CCCGAAACAGTAGCAGAGACAAG R: CTGGGGTCAGAGGAAGAGATAAAG	111	NM_213963
*SIRT1*	F: TGACTGTGAAGCTGTACGAGGAG R: TGGCTCTATGAAACTGCTCTGG	143	EU030283.2
*NRF1*	F: GCCAGTGAGATGAAGAGAAACG R: CTACAGCAGGGACCAAAGTTCAC	166	AK237171.1
*TFAM*	F: GGTCCATCACAGGTAAAGCTGAA R: ATAAGATCGTTTCGCCCAACTTC	167	NM_001130211
*ATP5B*	F: CATGAAGCAGGTGGCAGGTA	127	NM_001185142
	R: CAGACGAACACCACGACTCA		
*POLG*	F: CTTTGAGGTTTTCCAGCAGCAG	119	XM_005653521.1
	R: GCTCCCAGTTTTGGTTGACAG		
*SSBP1*	F: CTTTGAGGTAGTGCTGTGTCG	142	XM_005673118.1
	R: CTCACCCCTGACGATGAAGAC		
*COX4*	F: CCAAGTGGGACTACGACAAGAAC	131	Liu et al. [22]
	R: CCTGCTCGTTTATTAGCACTGG		
*CYCS*	F: TAGAAAAGGGAGGCAAACACAAG	154	Liu et al. [22]
	R: GGATTCTCCAGGTACTCCATCAG		
*CS*	F: CCTTTCAGACCCCTACTTGTCCT	127	Liu et al. [22]
	R: CACATCTTTGCCGACTTCCTTC		
*CuZn-SOD*	F: AACATGGTGGGCCAAAGGAT	136	NM_001190422.1
	R: CGGCCAATGATGGAATGGTC		
*ACTB*	F: TCTGGCACCACACCTTCT	114	DQ178122
	R: TGATCTGGGTCATCTTCTCAC		
*MT-CYB*	F: ATGAAACATTGGAGTAGTCCTACTATTTACC R: CTACGAGGTCTGTTCCGATATAAGG	149	NC_000845.1
*18S rRNA*	F: GGTAGTGACGAAAAATAACAATACAGGAC	141	NC_010448.3
	R: ATACGCTATTGGAGCTGGAATTACC		

*ACTB* β-actin, *COX* cytochrome c oxidase, *CS* citrate synthase, *CuZn-SOD* copper/zinc superoxide peroxidase, *MT-CYB* mitochondrially encoded cytochrome b, *CYCS* cytochrome c, somatic, *ATP5B* β subunit of mitochondrial H^+^-ATP synthase, *NRF1* nuclear respiratory factor 1, *PPARGC1A* peroxisome proliferator-activated receptor gamma coactivator 1α, *POLG* gamma DNA polymerase, *SIRT1* sirtuin 1, *SSBP1* mitochondrial single-strand DNA-binding protein, *TFAM* mitochondrial transcription factor A

### Quantitative real-time PCR

Total RNA was extracted from frozen muscle tissues using RNAiso Plus reagent (Takara, Dalian, China), and complementary DNA (cDNA) was synthesized from 0.5 μg of the total RNA using the PrimeScript™ RT reagent kit (Takara). Real-time quantitative PCR was carried out in the final volume of 10 ul containing 5 ul of SYBR® Premix Ex TaqTM II, 0.8 ul of the primer pair, 0.2 ul of ROX Reference Dye, 1 ul of cDNA template, and 3 ul of dH_2_O using the ABI Prism® 7900HT Sequence Detection System (Applied Biosystems, Foster city, CA, USA). The thermal cycling parameters comprised an initial denaturation step at 95 °C for 30 s, 40 cycles of PCR reaction at 95 °C for 5 s, and 60 °C for 34 s, followed by a dissociation step at 95 °C for 15 s, 60 °C for 1 min, and 95 °C for 15 s. To confirm specific product amplification, melt curve analysis was conducted. Primers for individual genes were designed using Primer Express 3.0 (Applied Biosystems) and are shown in Table 2. For normalization, *ACTB* was chosen as the reference gene since no variation in its expression was observed between treatments. The messenger RNA (mRNA) level of each target gene for SE group was set to 1.0.

### Immunoblotting analysis

Total protein was extracted from frozen muscle tissue using the protein extraction kit (Beyotime Biotechnology, Jiangsu, China) according to the manufacturer’s guide. The protein content of lysates was measured with the Pierce BCA protein Assay kit (Thermo, Waltham, MA, USA). Immunoblotting analysis was performed as previously described [29]. The primary antibodies included PPARGC1A (ab54481, Abcam, Cambridge, MA, USA), Sirt1 (sc-19857, Santa Cruz Biotechnology, CA, USA), CuZn-SOD (sc-271014, Santa Cruz Biotechnology), and β-tubulin (sc-9104, Santa Cruz Biotechnology). The density of bands was quantified using the Gel Doc XR System (Bio-Rad, Hercules, CA, USA) and then normalized to β-tubulin content. The normalized values were used for comparison of the expression of target protein between SE and LE groups.

### Statistical analysis

Results were presented as least square means with their standard errors. Data were analyzed using the MIXED procedures of Statistical Analysis System (version 8.1, SAS Institute, Inc.) with treatment, gender, and the two-way interactions as the fixed effect and with sow as the random effect. However, none of the gender and interactions were statistically significant. Therefore, they were removed from the model, and data from female and male fetuses were pooled together for analysis. The least square means were separated using the PDIFF option with the Turkey adjustment. The 2^−ΔΔCt^ method was used to analyze the relative changes in each gene expression [23]. Statistical significance was considered as *P* < 0.05, and a tendency towards difference was considered as *P* < 0.10.

## Results

### Pregnancy performance

On day 90 of pregnancy, the maternal body weight and fetal weight were reduced in LE group (*P* < 0.05; Table 3). However, no significant differences were observed for litter size and variation in fetal weight within a litter (as indicated by the coefficient of variation) between SE and LE groups (*P* > 0.05).

**Table 3:** Sow body weight, litter size, fetal weight, and variation in fetal weight within a litter on day 90 of pregnancy

Item	SE	LE	SEM	*P* value
Sow BW (kg)				
At insemination	72.7	72.9	0.9	0.840
At day 90 of pregnancy	127.9	117.0	1.9	0.002
Litter size	12.0	11.8	0.9	0.856
Fetal weight (g)	574.4	507.7	17.9	0.039
CV, %	14.86	12.72	1.48	0.345

*n* = 4 for each group

*SE* maternal standard-energy diet, *LE* maternal low-energy diet, *BW* body weight, *CV* coefficient of variation, values determined from fetal weights within each litter

### Serum concentrations of metabolites and hormone

As shown in Table 4, the glucose concentration tended to be reduced (*P* = 0.091) in umbilical vein serum of LE fetuses. Serum GH concentration was significantly decreased (*P* < 0.05) in LE fetuses compared with SE group, whereas maternal diet had no effect on the concentrations of triglyceride and insulin (*P* > 0.05).

**Table 4:** Concentrations of metabolites and hormones in fetal umbilical vein serum on day 90 of pregnancy

Item	SE	LE	SEM	*P* value
Glucose (mmol/l)	4.07	3.55	0.19	0.091
Triglyceride (mmol/l)	0.30	0.26	0.02	0.275
GH (ng/ml)	20.06	17.57	0.72	0.021
Insulin (pmol/ml)	134.98	130.16	2.80	0.227

*n* = 16 for each group

*SE* maternal standard-energy diet, *LE* maternal low-energy diet, *GH* growth hormone

### Mitochondrial DNA contents, CS activity, and the NAD^+^-to-NADH ratio

**Fig. 2:**
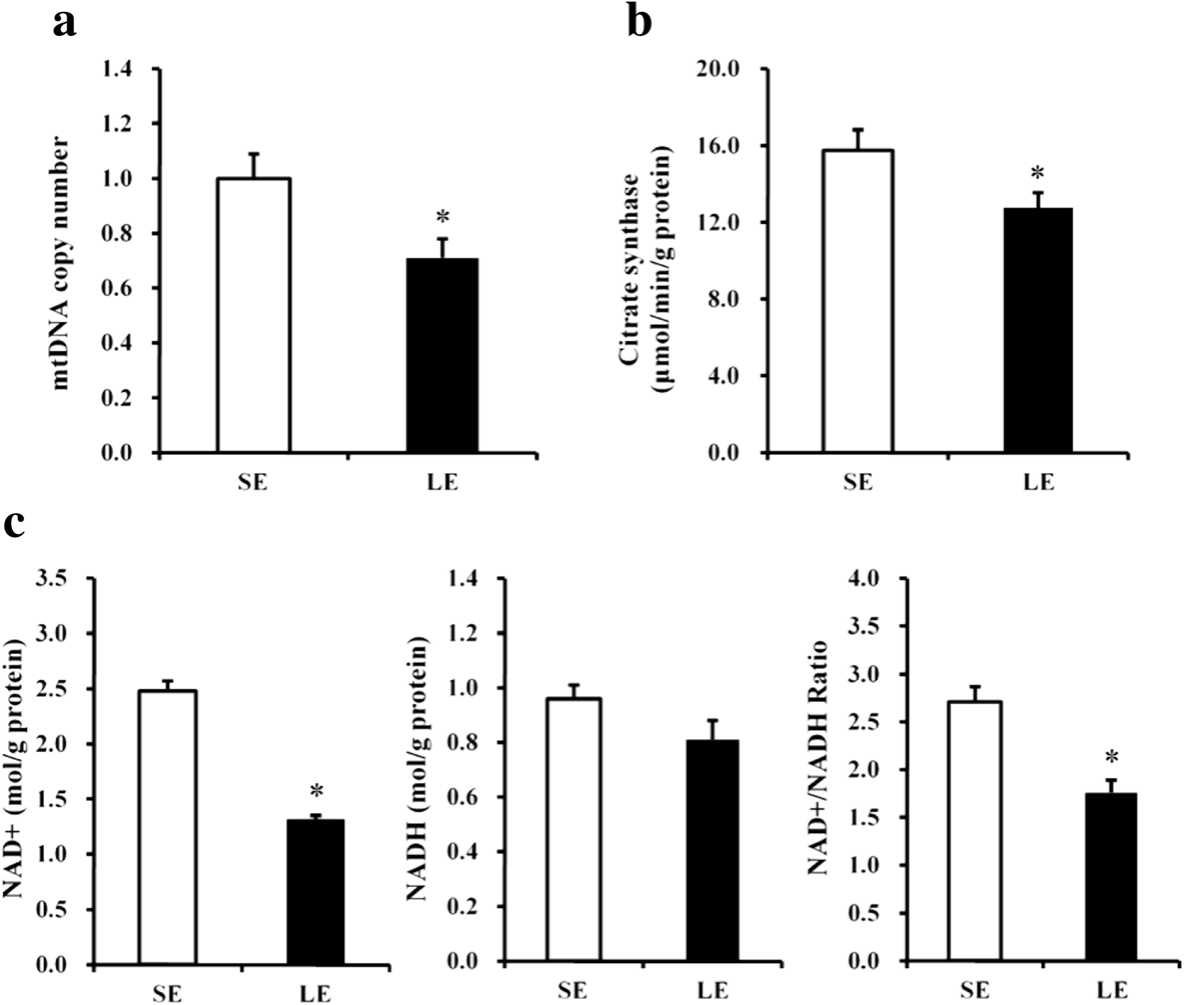
mtDNA copy number (**a**), citrate synthase activity (**b**), and the NAD^+^-to-NADH ratio (**c**) in the fetal longissimus muscles of SE and LE sows. Values are least squares means ± SEMs, *n* = 16. *SE* maternal standard-energy diet, *LE* maternal low-energy diet, *mtDNA* mitochondrial DNA. *P < 0.05 compared with SE group

As shown in Fig. 2a, the mtDNA copy number in the skeletal muscle was significantly lower (*P* < 0.05) in LE fetuses than in SE fetuses. Furthermore, CS activity was also reduced (*P* < 0.05) in LE fetuses (Fig. 2b). Though NADH level was not changed (*P* > 0.05), the NAD^+^ level and NAD^+^-to-NADH ratio were significantly decreased (*P* < 0.05) in the skeletal muscle of LE fetuses compared with SE fetuses (Fig. 2c).

### Expression of genes implicated in mitochondrial biogenesis and function

**Fig. 3:**
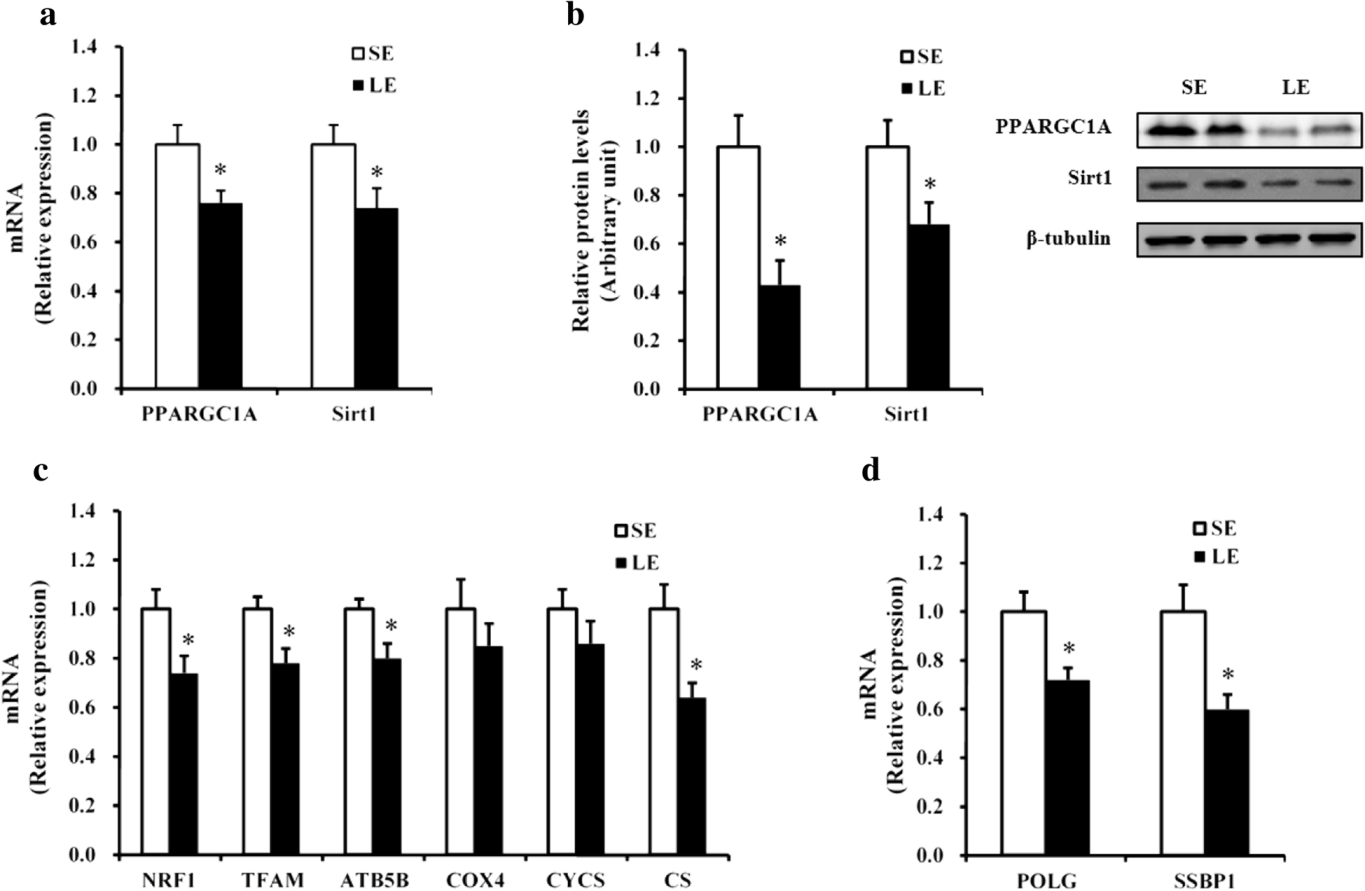
PPARGC1A and Sirt1 mRNA (**a**) and protein (**b**) levels and relative mRNA expression of the selected genes responsible for mitochondrial biogenesis and function (**c**) and mtDNA replication (**d**) in the fetal longissimus muscles of SE and LE sows. Values are least squares means ± SEMs, *n* = 16. *SE* maternal-standard energy diet, *LE* maternal low-energy diet. *P < 0.05 compared with SE group

To clarify the mechanisms involved in the reduction of mitochondrial density in the muscle of LE fetuses, we measured the expression of genes implicated in mitochondrial biogenesis and function. As shown in Fig. 3a, b, the mRNA and protein levels of PPARGC1A and Sirt1 in LE fetuses were decreased (*P* < 0.05) when compared with SE group. In addition, the mRNA abundance of nuclear respiratory factor 1 (NRF1), mitochondrial transcription factor A (TFAM), β subunit of mitochondrial H^+^-ATP synthase (ATP5B), and CS were significantly decreased in the skeletal muscle of LE fetuses as well (*P* < 0.05, Fig. 3c). Meanwhile, the mRNA abundance of gamma DNA polymerase (POLG) and single-stranded DNA-binding protein 1 (SSBP1) responsible for mtDNA replication were lower (*P* < 0.05, Fig. 3d) in LE fetuses.

### MDA level, CuZn-SOD gene expression, and antioxidant enzyme activities in the skeletal muscle

**Fig. 4:**
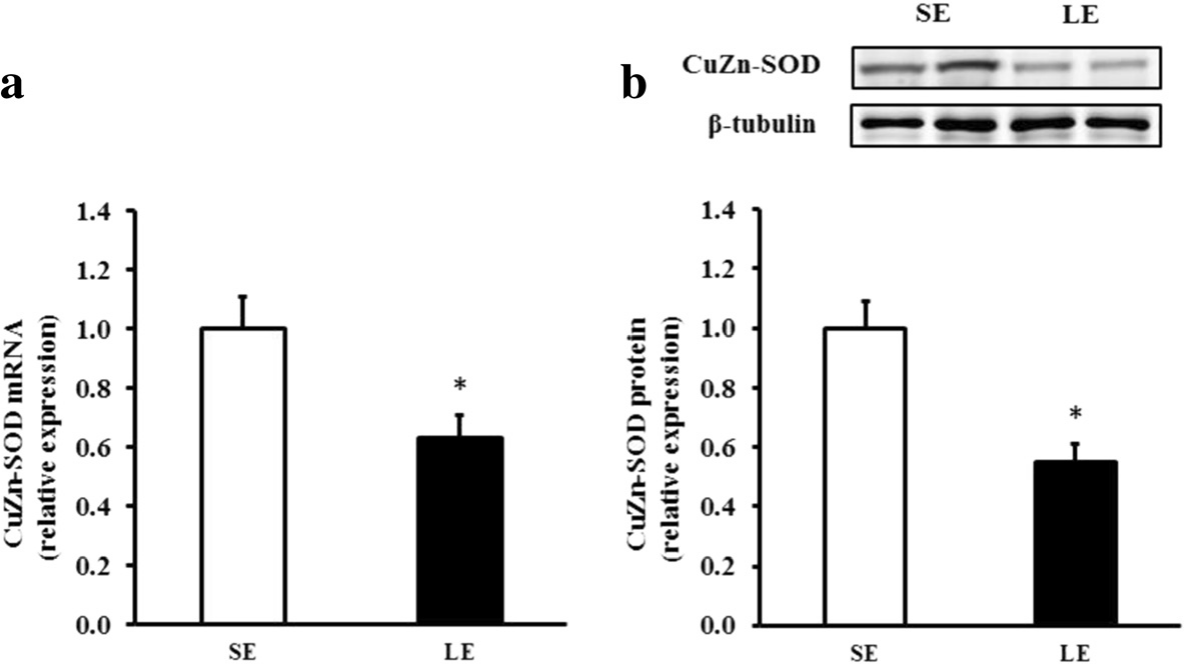
CuZn-SOD mRNA (**a**) and protein (**b**) expression in the fetal longissimus muscles of SE and LE sows. Values are least squares means ± SEMs, *n* = 16. *SE* maternal standard-energy diet, *LE* maternal low-energy diet. *P < 0.05 compared with SE group

We further measured the MDA level, CuZn-SOD expression, and the activities of three major antioxidative enzymes in the skeletal muscle to determine the effects of maternal energy restriction on reactive oxygen species (ROS) defense system. As shown in Table 5 and Fig. 4, the LE fetuses had markedly lower activities of SOD and CAT (*P* < 0.05) and mRNA and protein expression of CuZn-SOD (*P* < 0.05) in the skeletal muscle compared to the SE fetuses. No significant changes in MDA level and GPx activity were found between treatments (*P* > 0.05).

**Table 5:** Malondialdehyde content and antioxidant enzyme activities in the fetal longissimus muscle of SE and LE sows

Item	SE	LE	SEM	*P* value
MDA (nmol/mg protein)	2.18	2.05	0.13	0.468
SOD (U/mg protein)	32.39	28.59	0.51	0.008
GPx (U/mg protein)	40.32	36.17	2.05	0.169
CAT (U/mg protein)	8.35	6.41	0.45	0.006

*n* = 16 for each group

*SE* maternal standard energy diet, *LE* maternal low energy diet, *MDA* malondialdehyde, *SOD* superoxide dismutase, *GPx* glutathione peroxidase, *CAT* catalase

## Discussion

Epidemiological data demonstrate that low birth weight due to maternal malnutrition is associated with increased rates of metabolic diseases in adulthood [1, 11]. This study used the domestic pig as an animal model for the study of maternal nutrition and is of interest for human health and livestock science. Consistent with previous reports [15, 32], the maternal LE diets decreased fetal weight, which was linked to significantly lower serum GH concentration. Cumulative evidence have shown that mitochondrial dysfunction in the skeletal muscle is implicated in the developmental origins of metabolic syndrome [3, 43]. Mitochondria are inherited through the maternal lineage and easily affected by the environment because of its position outside the nucleus [37]. It was reported that maternal low-protein diets during gestation affect mtDNA transcription regulation through changing the DNA methylation and hydroxymethylation on the promoter of mtDNA in the liver of newborn piglets [15]. Previous studies in rodents also suggest that maternal nutritional stress causes changes to skeletal muscle mitochondrial function and oxidative metabolism that persist into adult offspring, increasing the risk of T2D [4, 21]. Moreover, because mitochondrial biogenesis accompanies skeletal myogenesis [34], and we previously reported that maternal LE diets attenuate fetal skeletal muscle development [48], thus, we hypothesized that the impairment of mitochondrial biogenesis may contribute to maternal LE diet-induced delayed skeletal myogenesis in pig fetuses. In the present study, we found that maternal LE diets during pregnancy decreased the mtDNA copy number in the skeletal muscle of LE fetuses, indicating a decrease in mitochondrial density. Similar results have been reported previously in rats’ offspring of dams fed with a low-protein diet [30, 41]. However, some studies have shown no consistent effects, and even advantageous effects, on mtDNA copy number when prenatal exposure to maternal low-protein diets in various tissues of different animal models, including the muscle in sheep [17] and muscle [4] and kidney [9] in rats, as well as fat in mice [18]. Thus, there are contradictory results on how maternal nutritional limitation during pregnancy affects mitochondrial biogenesis in the offspring. These findings imply that the time period and duration of treatment during pregnancy, the type and amount of nutrient changes, and the different animal models and tissues tested seem to be important factors resulting in variation between studies. To our knowledge, this is the first report regarding the effect of maternal LE diets on mtDNA content in the skeletal muscle of pig fetuses.

The decrease in muscle mtDNA content indicates disrupted mitochondrial biogenesis. The control of mitochondrial biogenesis is a complex biological process that needs the coordinated regulation of multiple transcriptional factors, including nuclear and mitochondrial-encoded genes [10]. It is well established that the NAD^+^-dependent deacetylase Sirt1 plays an important role in numerous fundamental cellular processes including gene silencing, DNA repair, and metabolic regulation [14]. In our study, the expression of Sirt1 at both the mRNA and protein levels was reduced in the skeletal muscle of LE fetuses. Because Sirt1 deacetylase activity is driven by NAD^+^ levels, we examined whether maternal LE diets suppress Sirt1 by altering the intracellular NAD^+^/NADH ratio. Supporting this hypothesis, the NAD^+^/NADH ratio was decreased in LE fetuses. However, NADH content was not changed by maternal LE diets, implying that, apart from the interconversion of NAD^+^ and NADH during redox reactions, the concentration of both partners of this redox pair may be controlled by other mechanisms as well. Previous study in astrocytes has shown that both NAD^+^ and NADH contents are controlled independently from each other [44]. Sirt1 deacetylates and activates PPARGC1A [10, 13], the master regulator of mitochondrial biogenesis that coactivates the NRF-1, which induce the expression of genes involved in mitochondrial biogenesis [46]. In the present study, we found that the mRNA and protein expression of PPARGC1A were reduced in the skeletal muscle of LE fetuses. Consistently, the mRNA expression of NRF1 were decreased in LE fetuses. With further investigation, the mRNA expression of TFAM, a direct regulator of mtDNA replication and transcription that could be regulated by PPARGC1A and NRF1 [46], was reduced in LE fetuses as well. Similar with previous reports in rats [30, 41], abnormal expression patterns of genes implicated in mtDNA biogenesis in the offspring liver or pancreatic islets of malnourished dams were observed. Additionally, because ATP5B is a critical molecule required for mitochondrial ATP synthesis [7], the decrease of mitochondrial biogenesis in LE fetuses was also reflected by down-regulated expression of ATP5B. Gamma DNA polymerase (POLG) and SSBP1 play a key role in mtDNA replication and repair [35]. As expected, we observed a decrease in POLG and SSBP1 in the skeletal muscle of LE fetuses. Together, these data show that maternal LE diets significantly reduced intracellular NAD^+^ levels and as a result decreased Sirt1 expression that subsequently decreased mtDNA replication and mitochondrial biogenesis in the skeletal muscle of LE fetuses.

Maternal malnutrition is associated with a depressed mitochondrial function and respiration in offspring [4, 9, 17]. In this study, although the mRNA expression levels of genes, such as COX4 and CYCS, responsible for mitochondrial function were not changed in LE fetuses, CS mRNA expression and activity were markedly decreased. Because it has been suggested a role for oxidative stress in skeletal muscle mitochondrial dysfunction [2], we speculate that oxidative stress may be implicated in undernutrition-associated mitochondrial alterations. ROS removal is regulated by many antioxidant enzymes, including SOD, GPx, and CAT [24]. In the present study, the mRNA and protein expression of CuZn-SOD and activities of SOD and CAT were reduced in the skeletal muscle of LE fetuses, suggesting the failure of ROS defense system, which is also an evidence of mitochondrial function changes [28]. However, muscle GPx activity was not changed by maternal LE diets. Thus, changes in the antioxidant system seem to occur in an enzyme-specific manner in prenatal energy-restricted fetal pigs. Similarly, previous study showed that maternal protein malnutrition significantly reduced SOD activity while GPx activity was not significantly affected in the liver of rat offspring [30]. Therefore, the enhanced oxidative stress may not explain completely the mitochondrial changes in the fetal muscle as a whole. Previous studies have shown that acute GH action promotes muscle mitochondrial function by stimulating mitochondrial oxidative capacity and transcript abundance of mitochondrial genes [38]. It is here shown that GH concentrations in umbilical vein serum of LE fetuses were reduced, which could, at least partly, explain the decreases in skeletal muscle mitochondrial biogenesis and function.

## Conclusions

**Fig. 5:**
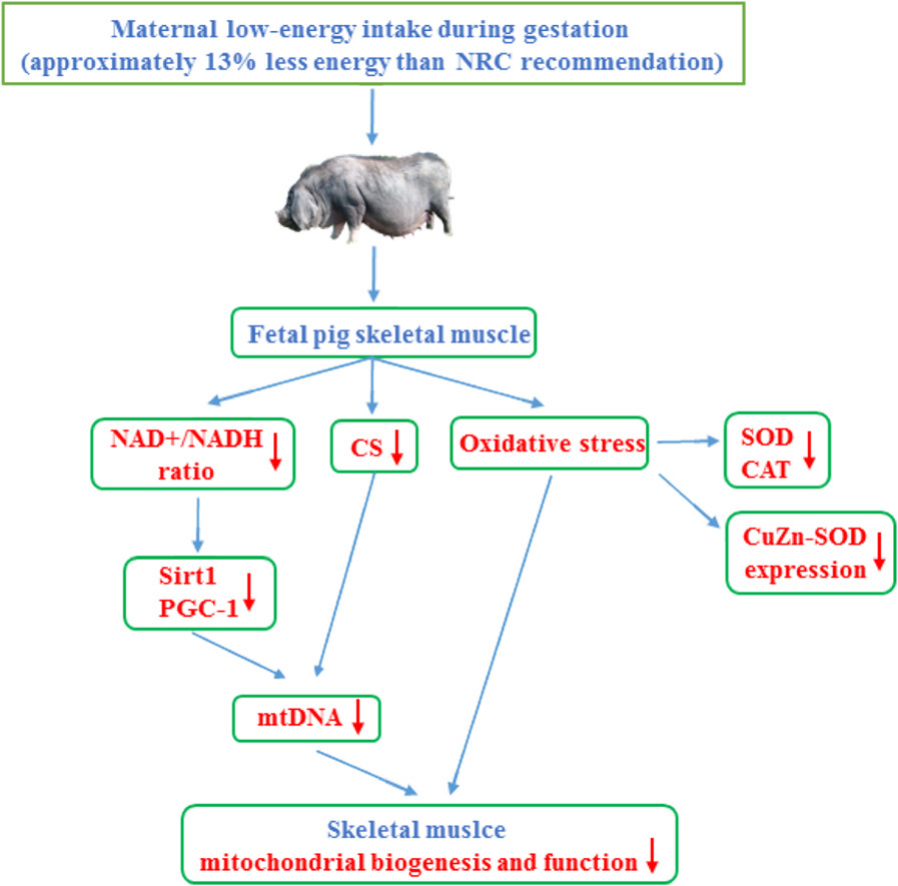
Diagram explains the effect of moderately decreased maternal energy intake during gestation on skeletal muscle mitochondrial biogenesis and function in fetal pigs

This study provides evidence that moderately decreased maternal dietary energy intake in pregnant sows during pregnancy impairs fetal development on day 90 of pregnancy, through decreased mitochondrial biogenesis and function in the skeletal muscle (Fig. 5). The mitochondrial changes during fetal life may further contribute to the disorder of energy homeostasis in adulthood. Consequently, understanding the molecular mechanisms underlying the effect of maternal undernutrition on fetal development may give access to useful knowledge regarding the onset of metabolic diseases.
